# Cardiovascular toxicity of tyrosine kinase inhibitors during cancer treatment: Potential involvement of TRPM7

**DOI:** 10.3389/fcvm.2023.1002438

**Published:** 2023-02-03

**Authors:** Qing Liu, Suyao Li, Yuran Qiu, Jiayu Zhang, Francisco J. Rios, Zhiguo Zou, Rhian M. Touyz

**Affiliations:** ^1^Department of Medical Oncology, Zhongshan Hospital, Fudan University, Shanghai, China; ^2^Cancer Center, Zhongshan Hospital, Fudan University, Shanghai, China; ^3^State Key Laboratory of Medical Genomics, Shanghai Institute of Hematology, National Research Center for Translational Medicine at Shanghai, Ruijin Hospital, School of Medicine, Shanghai Jiao Tong University, Shanghai, China; ^4^Research Institute of McGill University Health Centre, McGill University, Montreal, QC, Canada; ^5^Department of Cardiology, Renji Hospital, School of Medicine, Shanghai Jiao Tong University, Shanghai, China

**Keywords:** receptor tyrosine kinase, TRPM7, cardiovascular toxicities, cancer, tyrosine kinase inhibitors, magnesium, calcium, cation channel

## Abstract

Receptor tyrosine kinases (RTKs) are a class of membrane spanning cell-surface receptors that transmit extracellular signals through the membrane to trigger diverse intracellular signaling through tyrosine kinases (TKs), and play important role in cancer development. Therapeutic approaches targeting RTKs such as vascular endothelial growth factor receptor (VEGFR), epidermal growth factor receptor (EGFR), and platelet-derived growth factor receptor (PDGFR), and TKs, such as c-Src, ABL, JAK, are widely used to treat human cancers. Despite favorable benefits in cancer treatment that prolong survival, these tyrosine kinase inhibitors (TKIs) and monoclonal antibodies targeting RTKs are also accompanied by adverse effects, including cardiovascular toxicity. Mechanisms underlying TKI-induced cardiovascular toxicity remain unclear. The transient receptor potential melastatin-subfamily member 7 (TRPM7) is a ubiquitously expressed chanzyme consisting of a membrane-based ion channel and intracellular α-kinase. TRPM7 is a cation channel that regulates transmembrane Mg^2+^ and Ca^2+^ and is involved in a variety of (patho)physiological processes in the cardiovascular system, contributing to hypertension, cardiac fibrosis, inflammation, and atrial arrhythmias. Of importance, we and others demonstrated significant cross-talk between TRPM7, RTKs, and TK signaling in different cell types including vascular smooth muscle cells (VSMCs), which might be a link between TKIs and their cardiovascular effects. In this review, we summarize the implications of RTK inhibitors (RTKIs) and TKIs in cardiovascular toxicities during anti-cancer treatment, with a focus on the potential role of TRPM7/Mg^2+^ as a mediator of RTKI/TKI-induced cardiovascular toxicity. We also describe the important role of TRPM7 in cancer development and cardiovascular diseases, and the interaction between TRPM7 and RTKs, providing insights for possible mechanisms underlying cardiovascular disease in cancer patients treated with RTKI/TKIs.

## 1. Introduction

Receptor tyrosine kinases (RTKs) are a class of membrane-spanning cell-surface receptors that transmit extracellular signals through the membrane to trigger diverse intracellular signaling (1). In humans, 58 RTKs have been described that fall into 20 subfamilies (2).They all share a highly conserved structure, comprising a ligand-binding extracellular domain, an alpha-helical transmembrane domain and the tyrosine kinase domain (TKD) (3). Canonically, binding of ligands to RTKs induces dimerization and/or oligomerization of extracellular domains, resulting in activation of TKDs *via* trans-autophosphorylation and subsequent recruitment and activation of downstream signaling proteins. The activation of RTKs leads to phosphorylation and activation of numerous tyrosine kinases such as Abl, c-Src, Ras, PI-3K, JAK, and ALK, which regulatecellular processes, such as cell migration, differentiation, apoptosis, contraction, metabolism and survival (4). Activation of RTKs and TKs is critically involved in abnormal cell growth in cancer.

Abnormal expression and overactivation of RTKs, including vascular endothelial growth factor receptor (VEGFR), epidermal growth factor receptor (EGFR), and platelet-derived growth factor receptor (PDGFR) are associated with tumor invasion, metastasis, and tumor angiogenesis (5). Inhibiting these RTKs and their downstream signaling pathways reduce tumorigenesis and over the past 20 years there has been enormous interest in developing RTKI/TKIs as anti-cancer drugs (5). Despite favorable anti-cancer benefits, and prologed survival, TKIs are also accompanied by a profile of cardiovascular toxicities including hypertension, heart failure (HF) and arrhythmias (6). Understanding mechanisms of these side effects would improve the management of TKI-related cardiovascular toxicity and the clinical outcome.

The transient receptor potential melastatin-subfamily member 7 (TRPM7) possesses both ion channel and enzymatic functions. TRPM7 channel is permeable to divalent cations such as Zn^2+^, Mg^2+^ and Ca^2+^, and the α-kinase domain phosphorylates downstream substrates including annexin-1, eukaryotic elongation factor 2 (eEF2)'s cognate kinase (eEF2K), phospholipase Cγ2 (PLCγ2), myosin IIA, SMAD2, tropomodulin 1, myelin basic protein (MBP), cAMP response element binding protein (CREB), and RhoA (7–15). TRPM7 plays an important role in the cardiovascular system, regulating cardiac and vascular ion homeostasis, vascular smooth muscle cell function, vascular morphology and cardiac function. Abnormal TRPM7 activity has been implicated in hypertension, cardiac fibrosis, inflammation and atrial fibrillation (AF) (16–19), with TRPM7 downregulation promoting cardiovascular injury. On the other hand, aberrant expression of TRPM7 has been identified in various tumors, suggesting its significant involvement in tumorigenesis and cancer development (20–28).

In this review, we summarize the implications of RTK and TK inhibition in human cancers, with a focus on their cardiovascular toxicities. We also describe the important role of TRPM7 in cancer development and cardiovascular diseases, and the interaction between TRPM7 and RTKs, providing insights for possible mechanisms whereby anti-cancer drugs targeting RTKs induce cardiovascular toxicity.

## 2. RTKs, cancer, and cardiovascular toxicity

### 2.1. RTKs and oncogenesis

**Figure 1:**
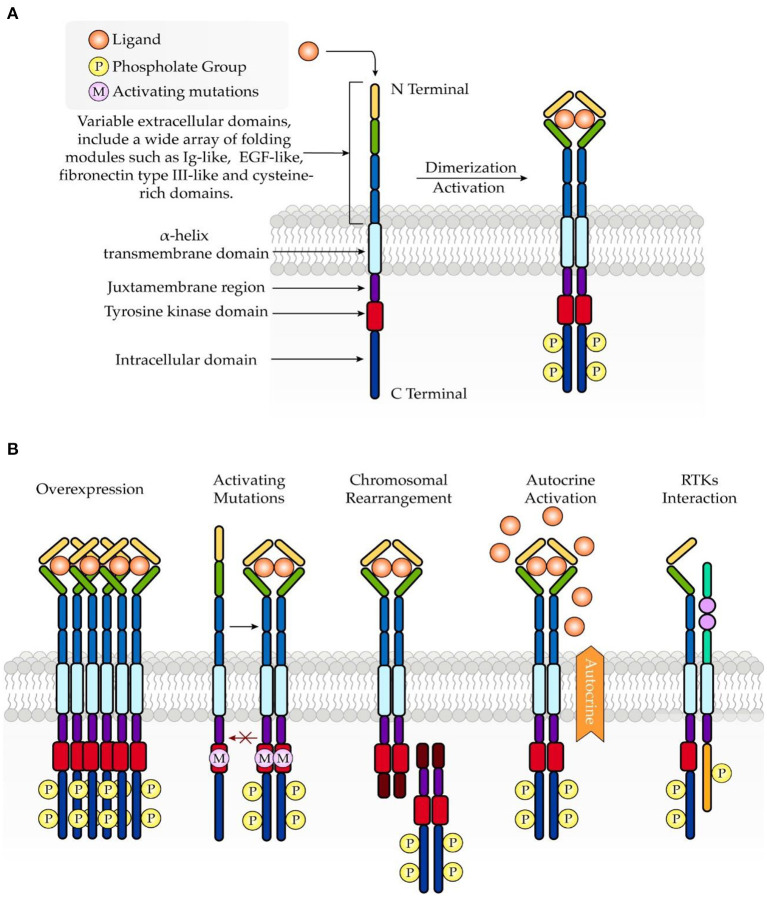
Schematic structure of RTKs and common mechanisms involved in oncogenesis. **(A)** RTKs consist of an extracellular ligand-binding domain, a transmembrane domain, and an intracellular tyrosine kinase domain. Upon ligand binding, RTKs undergo dimerization, which further leads to activation of the intracellular tyrosine kinase domain. **(B)** Abnormal activation of RTKs in turmor growth is associated with overexpression, mutations, chromosomal rearrangement, autocrine activation, and RTK interaction.

Under physiologic conditions, RTK activity is tightly controlled. However, dysregulated signaling through RTKs promotes an imbalance between cell proliferation and cell death, which is implicated in cancer development. Mechanisms underlying the aberrant activation of RTKs are associated with overexpression (29), mutations (30), chromosomal rearrangement (31), autocrine activation (32), and RTK interaction with other kinases, proteins, and signaling molecules (5) (Figure 1).

### 2.2. RTKs as therapeutic targets for cancer

TKIs are a class of pharmacologic agents that block multiple signal transduction pathways (33). In 2001, the US Food and Drug Administration (FDA) approved the first TKI imatinib, which targets the breakpoint cluster region protein- Abelson murine leukemia viral oncogene homolog (BCR-ABL) oncogene, and brought a revolutionary success to the treatment of chronic myeloid leukemia (CML) (34). To date, over 50 TKIs have been approved by the FDA. Major TKIs and their indications are listed in Table 1.

**Table 1:** TKIs approved by FDA in cancer treatment.

**Receptor**	**Class**	**Name of the drug**	**Trade name**	**Year of approval**	**Indication**
EGFR	TKI	Gefitinib	Iressa	2003	NSCLC (35–37)
		Erlotinib	Tarceva	2004	NSCLC/pancreatic cancer (38, 39)
		Afatinib	Gilotrif	2013	NSCLC (40)
		Dacomitinib	Vizimpro	2018	NSCLC (37, 41)
		Osimertinib	Tagrisso	2015	Medullary thyroid cancer (42)
		Vandetanib	Caprelsa	2011	NSCLC (39)
	mAb	Cetuximab	Erbitux	2004	CRC (43), head and neck cancer (44)
		Panitumumab	Vectibix	2006	CRC (45, 46)
		Necitumumab	Portrazza	2015	NSCLC (47)
HER2	TKI	Lapatinib	Tykerb	2007	Breast cancer (48, 49)
		Neratinib	Nerlynx	2017	Breast cancer (50, 51)
		Tucatinib	Tukysa	2020	Breast cancer (52)
	mAb	Trastuzumab	Herceptin	1998	Breast cancer (53), gastric or GEJ adenocarcinoma (54)
		Pertuzumab	Perjeta	2012	Breast cancer (55)
		Trastuzumab	Kadcyla	2013	Breast cancer (56, 57)
		Emtansine			
		Trastuzumab	Enhertu	2019	Breast cancer (58), gastric or GEJ adenocarcinoma (59)
		Deruxtecan			
		Margetuximab	Margenza	2020	Breast cancer (60)
VEGFR	TKI	Axitinib	Inlyta	2012	RCC (61)
		Cabozantinib	Cometriq	2012	MTC (62), RCC (63), HCC (64), DTC (65)
		Lenvatinib	Lenvima	2015	RCC (66), HCC (67)
		Pazopanib	Votrient	2009	RCC (68), soft tissue sarcoma (69)
		Regorafenib	Tafinlar	2012	CRC (70), GIST (71), HCC (72)
		Sorafenib	Nexavar	2005	RCC (73), HCC (74), or differentiated thyroid cancer (75)
		Tivozanib	Fotivda	2021	RCC (76)
		Sunitinib	Sutent	2006	GIST (77), RCC (78), or pancreatic neuroendocrine tumor (79)
		Vandetanib	Zactima	2011	Medullary thyroid cancer (80)
	mAb	Bevacizumab	Avastin	2004	Colon cancer (81), breast cancer (82), or ovarian cancer (83)
		Ranibizumab	Lucentis	2006	AMD (84) or diabetic macular edema (85)
		Ramucirumab	Cyramza	2014	NSCLC (86), gastric cancer (87), or CRC (88)
		Aflibercept	Eylea	2012	AMD (89), diabetic macular edema (85), or CRC (90)
PDGFR	TKI	Avapritinib	Ayvakittm	2020	GIST (91)
		Ripretinib	Qinlock	2020	GIST (92)
	mAb	Olaratumab	Lartruvo	2016	Soft tissue sarcoma (93)

NSCLC, non-small-cell lung cancer; GEJ, gastroesophageal junction; RCC, renal cell carcinoma; MTC, medullary thyroid carcinoma; HCC, hepatocellular cell cancer; DTC, differential thyroid cancer; CRC, colorectal cancer, GIST, gastrointestinal Stromal Tumors, AMD, age-related macular degeneration; TKI, tyrosine kinase inhibitors; mAb, monoclonal antibody.

### 2.3. Cardiovascular toxicity associated with tyrosine kinase inhibition

#### 2.3.1. VEGFR inhibitors and cardiovascular toxicity

VEGF signaling plays a critical role in angiogenesis, cell proliferation and survival. Following advances in knowledge about the role of angiogenesis in promoting tumor growth (94, 95), multiple clinical trials demonstrated that VEGFR inhibitors yield incremental improvements in outcomes for a variety of solid tumors. However, the increasing use of these agents is also associated with a wide spectrum of side effects, most frequently related to cardiovascular toxicity, which might be linked to direct effects of VEGF inhibitors on the vasculature (96).

##### 2.3.1.1. Hypertension

Hypertension is considered as the main cardiovascular side effect of VEGFR-TKIs. Almost every trial reports treatment-induced blood pressure elevation and up to 80% of patients develop hypertension, either *de novo* or worsening of previously controlled hypertension (97). As outlined in a meta-analysis including 77 VEGF inhibitors, severe hypertension occurred in 7.4%, cardiac dysfunction in 2.3%, arterial thromboembolism in 1.8%, and cardiac ischemic in 1.7% of patients, and there was no significant difference in cardiovascular risk between anti-VEGF monoclonal antibody and TKIs (98). Molecular mechanisms underlying the development of hypertension during VEGFR-TKI therapy remain unclear. However, many studies have shown that VEGFR inhibitors reduce the level of vasodilators, including nitric oxide (NO) and PGI2, which are crucial in the development of hypertension. In patients treated with VEGFR-TKI, plasma levels of NO and its metabolites are decreased, but return to baseline following withdrawal (99). Also, VEGFR-TKI therapy is associated with an elevated level of endothelin-1 (ET-1, a potent vasoconstrictor) (100). ET-1 receptor antagonists have been shown to be effective to treat VEGFR-TKI-associated hypertension in a pre-clinical study suggesting the involvement of ET-1 in VEGFR-TKI-related hypertension (101).

##### 2.3.1.2. Thrombosis

VEGFR inhibition is known to cause both arterial thrombosis event (ATE), particularly cardiac ischemia, and venous thromboembolism (VTE). The risk of ATE associated with anti-VEGF TKIs is greater than that of VTE, with an incidence generally <3% (102, 103). A meta-analysis of 19 randomized controlled trials including 9,711 patients treated with anti-VEGF TKIs showed a significantly increased risk of developing ATE when compared with controls (OR 2.26, 95% CI: 1.38–3.68, *p* = 0.001), with cardiac ischemia/infarction (67.4%) as the most common events for ATE (104). However, another meta-analysis of 7,441 patients from 17 phrase II/III trials reported no difference in the relative risk of VTE for anti-VEGF TKIs compared with controls (105). Several mechanisms have been proposed to account for the thromboembolic events of anti-VEGF therapy. Apart from the facilitation of endothelial cell proliferation and survival, VEGF activity is crucially involved in the maintenance of vascular integrity (106, 107). Hence, blockage of VEGF signaling impairs the integrity and regenerative capacity of endothelial cells, subsequently leading to thrombosis. Moreover, decreased level of PGI2 and NO related to anti-VEGF therapy creates a procoagulant environment in the vessel wall, predisposing patients to thromboembolic events (106).

##### 2.3.1.3. Heart failure

Meta-analysis of trials of VEGFR-TKIs (sunitinib, sorafenib, pazopanib, axitinib, vandetanib, cabozantinib, ponatinib and regorafenib) including 10,647 patients demonstrated a pooled incidence of asymptomatic HF of 2.4% and symptomatic HF of 1.2%. Notably, there was no apparent difference in the risk of cardiovascular toxicity between the relatively specific VEGFR-TKIs (e.g., axitinib) and those targeting against a broader range of tyrosine kinases (e.g., sunitinib, sorafenib, and pazopanib) (108). Mechanisms underlying VEGFR-TKI-associated HF appear to be highly relevant to the cardiac afterload increased by endothelial dysfunction and hypertension (109). Mitochondrial dysfunction and cytochrome C-induced apoptosis might also be important, which are caused by the on-target VEGF signaling inhibition of the PI3K-AKT pathway (110). Moreover, inhibition of not only angiogenesis but also other off-targets, such as PDGFR and the adenosine monophosphate-activated protein kinase (AMPK), might be implicated in potential mechanisms that lead to HF (6).

##### 2.3.1.4. QT prolongation

The incidence of QT prolongation associated with VEGFR-TKIs varies widely among individual drugs. Vandetanib has the highest incidence and the most significant prolongation, with up to 8% of patients exhibiting a corrected QT (QTc) interval duration of >500 ms (111). Meta-analysis of VEGFR-TKI related clinical trials demonstrated an incidence of 4.4% of all-grade QT prolongation when compared to non-TKI therapy (112). The exact mechanism of QT prolongation from anti-VEGF TKIs is unclear, while it has been hypothesized that these TKIs might interact with the human ether-a-go-go-related gene (hERG) potassium channels, predisposing to QT prolongation (113). Of major significance, hypomagnesemia, possibly due to abnormal TRPM7 activity, is a major cause of long QT syndrome (114).

#### 2.3.2. EGFR inhibitor and cardiovascular toxicity

EGFR is a cell surface protein that binds to epidermal growth factor, thus inducing receptor dimerization and tyrosine autophosphorylation leading to cell proliferation. Mutations in this gene have been associated with a number of cancers, including adenocarcinoma of the lung (115), glioblastoma and epithelial tumors of the head and neck (116). Based on the FDA Adverse Events Reporting System (FAERS), a public database that contains nearly 17 million (adverse event) AE reports, EGFR TKIs have been associated with HF and cardiac arrhythmias such as atrial fibrillation and QT prolongation (117). In a retrospective cohort study of 123 patients with advanced non-small cell lung cancer (NSCLC) who received Osimertinib, the first third-generation EGFR-TKI, the incidence of cardiac AEs was 4.9% (118).

##### 2.3.2.1. Heart failure

A meta-analysis of 10 randomized clinical trials involving 12,000 patients treated by trastuzumab, a monoclonal antibody targeting HER2 (the family member of EGFR), indicated that the incidence of asymptomatic decline of left ventricular ejection fraction (LVEF, normal range 50–70%) and symptomatic HF was 7.5 and 1.9%, respectively (119). A case-control study of 53 patients receiving HER2-targeted therapy further found that an LVEF <55% at any surveillance timepoint was associated with higher risk for HF, suggesting that baseline cardiac function might be an important factor that determines the cardiac outcome (120). Additionally, an increasing number of case reports have demonstrated significant HF induced by Osimertinib in patients with lung cancer, a problem that could be complicated by the coincidence of QT prolongation (121–123). In animal studies, Threadgill et al. found that wild-type mice displayed cardiac dysfunction and increased cardiac fibrosis after 3-month exposure to EGFR-TKIs (124). Intriguingly, female mice exhibited increased cardiac adverse effects, suggesting that sex might influence the susceptibility to TKI-mediated toxicity (124). Furthermore, taking advantages of the myoblast cell line H9c2 and *in vivo* rat cardiomyocytes, Korashy et al. showed that Gefitinib, a selective inhibitor of EGFR, induces cardiovascular toxicity through modulating the cardiac PTEN/AKT/FoxO3a pathway and the formation of CYP1A1-mediated reactive metabolites (125).

##### 2.3.2.2. Cardiac arrhythmia

In a retrospective study based on the world health organization (WHO) pharmacovigilance database VigiBase, among 98,765 adverse reactions reported with NSCLC-targeted therapies including EGFR-TKIs, 1,783 (1.8%) were cardiac arrhythmias (126). The most frequently reported cardiac arrhythmia associated with EGFR-TKIs is QT prolongation. Rociletinib, a third-generation EGFR-TKI targeting common EGFR-activating mutations, was found to increase the risk of corrected QT prolongation compared to chemotherapy (6.7 vs. 0%) in patients with advanced or metastatic NSCLC (127). Case reports showed that Osimertinib induced QT prolongation in patients with lung cancer, while discontinuation of the drug led to the alleviation of prolonged QT interval (121, 123, 128). The scenario is further complicated by the concurrence of HF, while whether there is a casual link remains unclear (121, 123). These studies also recommend that careful monitoring of electrocardiogram (ECG) and serum potassium, a cation that importantly affects QT interval, is required in neoplastic patients receiving EGFR-TKIs therapy (128). It is also worth noting that several clinical studies failed to provide evidence supporting any correlation between anti-EGFR therapy and cardiac abnormalities including QT prolongation, and thus future adequately powered clinical trials are still required (129, 130).

#### 2.3.3. PDGFR inhibitor and cardiovascular toxicity

PDGFR signaling pathway plays a critical role in promoting cardiomyocyte proliferation and heart regeneration (131). Various TKIs that inhibit VEGFR/PDGFR were shown to induce significant cell death in human induced pluripotent stem cell-derived cardiomyocytes (hiPSC-CMs) (132). Additionally, in an animal model of myocardial infarction, PDGF gene transfer was able to improve left ventricular function (133). In considering the potential cardioprotective effect of PDGF, it is not surprising that anti-PDGFR therapy in cancer patients was associated with cardiac AEs. Sunitinib, a TKI with multiple targets including PDGFR, was reported to induce blood pressure elevation and LVEF reduction (134). Similarly, Dasatinib, a multitargeted TKI, was shown to prolong ventricular effective refractory period and impair left ventricular mechanical function in dogs at a low-dose administration (135). Moreover, in a recent Japanese cohort study, cardiotoxic AEs including congestive HF, pericardial effusion and QT prolongation were frequently reported in patients with chronic myeloid leukemia (CML) and gastrointestinal stromal tumor (GIST) (136). However, findings of these studies should be interpreted carefully, since the observed cardiac effects might be attributed to other targets of the treatment, while available evidence regarding PDGFR-selective TKIs still lacks.

## 3. TRPM7 as a potential contributor to RTKI-induced cardiovascular toxicity

### 3.1. TRPM7 in the cardiovascular system

**Figure 2:**
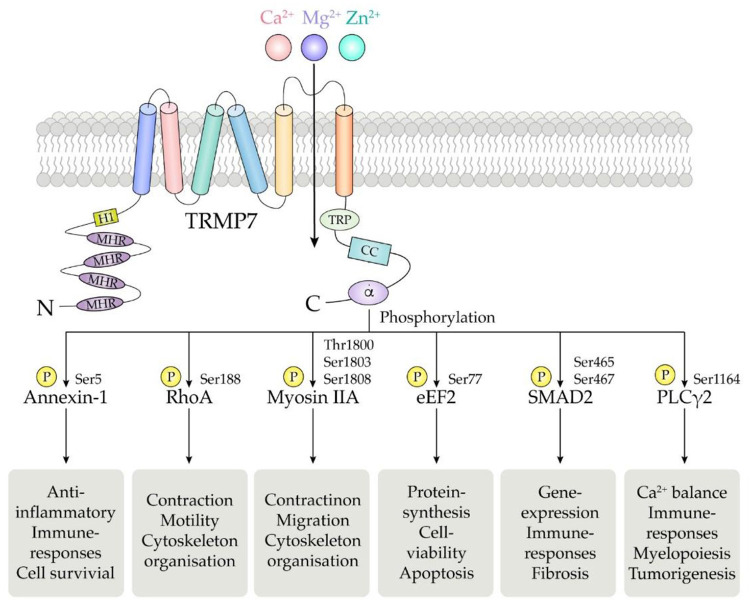
Schematic structure of the TRPM7 chanzyme. It consists of six transmembrane segments with the channel pore formed between segment 5 and 6. TRPM7 channel is permeable to divalent cations including Mg^2+^, Ca^2+^, and Zn^2+^.The α-kinase domain is able to phosphorylate diverse downstream substrates such as annexin-1, RhoA, myosin IIA, eEF2, SMAD2, and PLCγ2, at specific phosphorylation residues.

TRPM7 human gene is located on the long arm of chromosome 15, consisting of 19 exons and encoding a 1,863 amino acid protein with a molecular weight of 210 kDa (137). The basic structure of TRPM7 consists of N-terminal hydrophobic region (H1) and four Melastatin Homologous Regions (MHR), six transmembrane segments, and C-terminal transient receptor potential (TRP) region followed by the coiled coil (CC) domain connecting loop, serine/threonine-rich domains and an α-type kinase domain (Figure 2) (4).

TRPM7 expression has been confirmed in the most abundant cell types of the cardiovascular system including VSMCs, endothelial cells (ECs) and cardiomyocytes (16, 138, 139). In 2005, we were amongst the first to identify and characterize TRPM7 in VSMCs, where the chanzyme acts as a functionally important regulator of Mg^2+^ homeostasis and cell growth (16). We recently further showed that TRPM7 is membrane-bound in VSMCs, mediating Mg^2+^ and calcium (Ca^2+^) influx and exerting effects on cell migration and proliferation (17). TRPM7 was found to be a contributor to the development of a proliferative phenotype of VSMCs trigged by angiotensin II (Ang II), and vascular calcification induced by phosphate (140, 141). In ECs, Inoue and Xiong discovered the TRPM7-like outward rectifying currents by whole-cell patch-clamp experiments (138). The physiological function of TRPM7 in ECs is more likely to associate with its Mg^2+^ permeability, since silencing TRPM7 mimics the effects of Mg^2+^ deficiency on cell behavior and Mg^2+^ regulates endothelial barrier functions through TRPM7 (142, 143). Additionally, endothelial functions such as cell adhesion and tube formation are negatively regulated by TRPM7 with mechanisms involving the extracellular signal-regulated kinase (ERK1/2) pathway (144). TRPM7 and its sister homolog TRPM6 are also observed in cardiomyocytes from all chamber walls of human hearts (139). Cardiac TRPM7 influences cardiac action potentials in a Mg^2+^-sensitive manner, while TRPM7 deletion in embryonic myocardium disrupts cardiac automaticity *via* the regulation of Hcn4 expression (145, 146).

TRPM7 activity is regulated by various vasoactive agents such as Ang II, aldosterone, bradykinin and C-type natriuretic peptide (CNP) (16, 147–149). Ang II enhances TRPM7 protein expression through Ang II type 1 receptor-mediated ERK1/2 signaling, which contributes to phenotypic change and proliferation of VSMCs (140). Chronic treatment with aldosterone upregulates the plasma membrane expression of TRPM7 in HEK cells, a process occurring *via* a mineralocorticoid receptor (MR)-dependent genomic signaling cascade involving serum- and glucocorticoid-regulated kinase 1 (SGK1) and a functional TRPM7 α-kinase domain (148). Bradykinin, a known vasodilator, was found to mediate the expression of TRPM7 and its kinase substrate annexin-1 in VSMC *via* molecular mechanisms involving phospholipase C (PLC), protein kinase C (PKC) and c-Src (149). Furthermore, C-type natriuretic peptide (CNP), a peptide produced by the vascular endothelium, has recently been shown to affect TRPM7-mediated Ca^2+^ entry in chondrocytes and stimulate bone growth *via* activating natriuretic peptide receptor 2 (NPR2) (147).

Growing evidence indicates an impoartnt role for TRPM7 in cardiac development (146). TRPM7 plays an indispensable role for myocardial proliferation during early cardiogenesis, as *TRPM7* deletion before embryonic day 9 led to congestive heart failure and death by embryonic day 11.5 (150). These findings highlight the importance of TRPM7 in the integrity and function of the cardiovascular system.

### 3.2. TRPM7 and cardiovascular diseases

#### 3.2.1. Cardiac fibrosis

Cardiac fibrosis is defined as the excessive accumulation of fibrillar extracellular matrix in the cardiac interstitium, a pathological process contributing to various heart diseases including HF, myocardial infarction, dilated and ischemic cardiomyopathies and arrhythmias (151). We demonstrated that TRPM7 deficiency is associated with cardiac dysfunction, inflammation and fibrosis in mice by Mg^2+^ dependent effects (17). Intriguingly, TRPM7 was observed to contribute to cardiac fibrosis induced by Ang II and hydrogen peroxide (H_2_O_2_) (152, 153). In these studies, 2-APB, a pharmacological non-specific inhibitor of TRPM7, was shown to attenuate cardiac fibrosis *via* effects on cardiac fibroblasts (152, 153). Given the important role of TRPM7 played in cardiac fibrosis, several studies have investigated TRPM7 as the potential therapeutic target. Tang et al. showed that Astragaloside IV, an important constituent of traditional Chinese medicine, inhibits cardiac fibrosis through modulating TRPM7 (154). Moreover, it was also shown that sacubitril, a drug well-known for treating heart failure, ameliorates cardiac fibrosis by acting on fibroblasts and cardiomyocytes *via* inhibiting TRPM7 channel (155).

#### 3.2.2. Hypertension

Hypertension is a well-known major risk factor for cardiovascular diseases and exerts negative effects on healthy longevity (156). Direct evidence supporting TRPM7 involvement in the development of hypertension was first demonstrated in Ang II-induced hypertension, where the development of hypertension was amplified in TRPM7-deficent mice. This was associated with impaired endothelial function and amplified cardiac remodeling and left ventricular dysfunction (18). On the other hand, it was shown that TRPM7 inhibition by pharmacologic agents reduced hypertension induced by leptin. (157). Leptin receptor and TRPM7 colocalized in glomus cells of carotid bodies, where leptin regulates blood pressure through acting on TRPM7 (157). Furthermore, calpain, the substrate of TRPM7 kinase, might also play an important role in the development of hypertension. Calpain acts as downstream mediators in Ang II-induced cardiovascular remodeling, while calpastatin, a calpain-specific inhibitor, was able to prevent Ang II-dependent cardiac hypertrophy and perivascular inflammation (158). In addition, TRPM7 might contribute to hypertension *via* its Mg^2+^ permeability. Despite inconsistent findings to support the correlation between serum Mg^2+^ and hypertension, Mg^2+^ supplementation at a dose of 368 mg/d for 3 months has been shown to lower blood pressure in adults (159–161).

#### 3.2.3. Cardiac arrhythmia and ischemic heart disease

In the last decade, increasing evidence have established a link between TRPM7 and atrial fibrillation (AF), which is most commonly sustained arrhythmia and a major cause of morbidity and mortality (19, 162–164). Elevated TRPM7 expression has been observed in atrial myocytes and peripheral blood from patients with AF (162, 164). Yue et al. further showed that TRPM7 is the major Ca^2+^ permeable channel in human atrial fibroblasts, which might contribute to atrial fibrosis in human AF (19). These findings suggest that TRPM7 is associated with the pathogenesis of AF, which might be related to its cation permeability property. TRPM7 is highly expressed in sinoatrial node, where it influences diastolic membrane depolarization and automaticity, suggesting a possible role of TPRM7 in sick sinus syndrome and atrioventricular block (146). TRPM7 might also be implicated in ventricular arrhythmias, since cardiac TRPM7 deletion in mice is associated with a high risk of developing cardiomyopathy, characterized by impaired repolarization and ventricular arrhythmias (150). Importantly, attention has also been drawn to the involvement of TRPM7 in ischemic heart disease (IHD), since increased expression of TRPM7 and TRPM6 was observed in cardiac biopsies from patients with IHD (139). Consistently, in a murine model of myocardial infarction, TRPM7 expression was remarkably upregulated in cardiac fibroblasts, accompanied by enhanced Ca^2+^ influx (165).

## 4. Cross-talk between TRPM7, Mg^2+^, and RTKs

**Figure 3:**
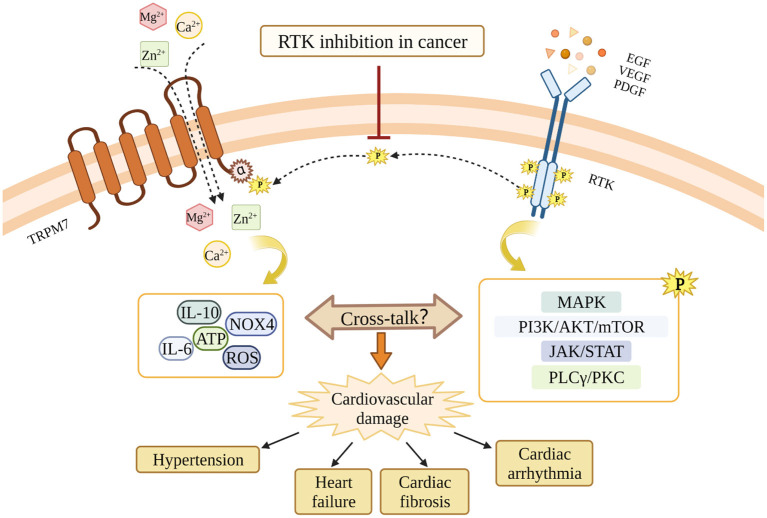
Potential cross-talk between RTKs and TRPM7 and the potential involvement in RTKs inhibition-related cardiovascular toxicity. RTKs inhibition blocks the interaction between RTKs and TRPM7, and leads to TRPM7 dysfunction. Through mechanisms involving TRPM7, RTKs inhibition might cause cardiovascular damages in cancer patients. The MAPK pathway, the PI3K/AKT/mTOR pathway, the JAK/STAT pathway, and the PLCγ/PKC pathway are downstream signaling cascades activated by RTKs. The dashed line indicates the possibility that RTKs and TRPM7 interact. This still awaits confirmation.

Intracellular Mg^2+^, which is regulated by TRPM7 cation channel activation, is an essential cation and second messenger involved in tyrosine kinase signaling and regulation of RTKs (166). Mechanisms whereby Mg^2+^ regulates the activation of RTKs are elusive, however the current concept of “two metal catalysis” shows the requirement of two Mg^2+^ ions for proper kinase phosphorylation. In physiologic conditions, the ligand-receptor interaction induces binding of the first Mg^2+^ ion to the kinase, which allows the binding of the second Mg^2+^ leading to a proper signal propagation of the TK. This complex signaling pathway is deficient in conditions of Mg^2+^ deprivation (167). Moreover, affinity analysis showed that the binding-free energies of ATP to target enzymes are lower in the presence of Mg^2+^ ions than those in the absence, suggesting that Mg^2+^ enhances the binding affinities of ATP to the protein kinases (168) (Figure 3).

In renal distal convoluted tubule (DCT), EGF increases TRPM6 activity and surface expression, while in cancer patients receiving anti-EGFR treatment, serum level of Mg^2+^ was significantly decreased (169–171). In a similar fashion, EGF through binding to EGFR and activating downstream signaling, regulates cell membrane proteins expression and currents of TRPM7 (21, 172). PDGF was shown to dramatically enhance the gene and protein expression of TRPM7 in a time-dependent manner in hepatic stellate cells (HSCs), and PDGF-induced cell proliferation was prevented by TRPM7 inhibition (173). Consistently, Xu et al. found that carvacrol, a pharmacological inhibitor of TPRM7, attenuated the activation and proliferation of HSCs induced by PDGF through TRPM7-meidated cellular signaling involving the mitogen-activated protein kinases (MAPK) (174). In a human osteoblast cell line, PDGF upregulated TRPM7 expression after 4 h of treatment, an effect that could be sustained for a 24 h-period (175). The regulation of TRPM7 by PDGF was further shown to importantly modulate Mg^2+^ influx, and proliferation and migration of human osteoblasts (175). Moreover, nerve growth factor (NGF) *via* its receptor TrkA, a family member of RTKs, regulated the outward-rectifying TRPM7-like currents in hippocampal neurons through a phospholipase C (PLC)-dependent manner (176). Taken together, these studies have highlighted a critical regulation of TRPM7 by the RTKs signaling, which is associated with important biological effects in various cell types.

It is worth noting that most evidence connecting RTKs and TRPM7 were from non-cardiovascular studies. The direct evidence demonstrating a link between TKIs and TPRM7 in the cardiovascular system was from our previous study and this link was primarily functional (177). We showed that in VSMCs, EGF promotes Mg^2+^ influx through the TRPM7 channel and consequently regulates VSMCs function and vascular morphology. Of importance, the effects of EGF on TRPM7 and VSMCs were attenuated by gefitinib, a TKI that exclusively targets EGFR. In addition, we also found that in VSMCs from TRPM7 kinase-deficient mice (TRPM7^+/Δ*kinase*^) and aortic tissues from TRPM7-kinase dead (TRPM7^R/R^) mice, EGFR expression and EGFR phosphorylation (Y845) were reduced, respectively, supporting a significant cross-talk (177). Moreover, our study demonstrated that EGF/EGFR was able to mediate the kinase activity of TRPM7 in VSMCs, due to the observation that EGF enhanced TRPM7 phosphorylation at Ser 1511 (177).

Experimental treatment with the EGFR inhibitor erlotinib causes hypomagnesemia that gradually increases after 3 weeks of treatment, suggesting a cumulative chronic effects on the Mg^2+^ handling by kidneys and intestines (178). Oxidative stress is another important mechanism that might be responsible for reduced activity of Mg^2+^ channels observed after EGFR inhibition. Erlotinib activated Nox4, a NADPH oxidase highly expressed in the cardiovascular system that generates H_2_O_2_ (179), and it was previously demonstrated that H_2_O_2_ cause inhibition of TRPM6 and TRPM7 activities and subsequently reduction in Mg^2+^ (180, 181). However, it is still elusive whether these important mechanisms are observed in cells from the vasculature.

Vascular dysfunction in hypertension resembles the vascular phenotype in aging, with hypertension being defined as a condition of premature vascular aging (182). Cellular and experimental studies demonstrated that TRPM7-deficiency is associated with increased molecular markers of senescence, including p16, WRN and phosphorylation of P66Shc (183). Importantly, expression of these markers is increased in experimental hypertension, induced by infusion with Ang II or aldosterone, chronic kidney ischemia and pulmonary arterial hypertension (182). Important pathologic implications of the TRPM7-Mg^2+^ axis deficiency is associated with cardiovascular inflammation by mechanisms dependent on macrophage infiltration to cardiac tissues leading to increased galectin-3, IL-6, IL-10, phosphor-P66Shc and reduced phosphor-Stat3 leading to cardiac fibrosis and diastolic dysfunction (17). Additionally, TRPM7 is downregulated in VSMC from PAH patients and in experimental models, effects that were exacerbated by waixenicin A, a TRPM7 inhibitor. Intracellular mechanisms involved MEK/ERK pathway (184).

Another important factor that should be taken into consideration is that TRPM7 is highly permeable to Zn^2+^. Hence, downregulation of TRPM7 induced by TKI might affect intracellular concentration of Zn^2+^. Clinical investigations showed reduced serum Zn^2+^ as adverse effect of EGFR inhibition and Zn^2+^ supplementation was able to reduce dermatitis these patients (185). Of importance, Zn^2+^ is a potent antioxidant and its deficiency is associated with mitochondrial and endoplasmic reticulum (ER) stresses, increased ROS production and dysregulation of cellular metabolism (186). Clinically, Zn^2+^ deficiency is associated with high incidence of cardiovascular diseases, including hypertension and diabetes (187). Mechanisms underlying these effects are still elusive and might involve oxidative stress and inflammatory response.

Mg^2+^ deficiency is directly associated with risk for diabetes development. Physiologically, Mg^2+^ is a co-factor of the ATPase that limits the opening of the ATP-sensitive potassium channels, leading to increase in calcium influx and results in insulin release. Therefore, low Mg^2+^ reduces ATPase activity and consequent hyperactivity of the ATP-sensitive potassium channels (KATP), inhibiting Ca^2+^ influx resulting in defective insulin secretion (188). Furthermore, hypomagnesemia interferes with optimal binding of insulin to receptor and because IRS are TK receptor, reduced in Mg^2+^ interferes with intracellular signaling pathways mediated by IRS1 and IRS2 activation (167, 189). Of importance, a randomized clinical trial involving 1,122 subjects showed that hypomagnesemia is associated with the development of impaired glucose tolerance and type 2 diabetes (190).

It should be highlighted that while it is clear that there is important cross-talk between RTK/TK and TRPM7 at the functional level, direct interacting p-tyr sites between RTKs/TKs and TRPM7 still awaits confirmation. However, there is growing evidence that both TRPM6 and TRPM7 possesses phosphorylation sites, which potentially may link to the RTK/TK pathway (14, 191). Moreover, adaptor proteins may act as links between the systems.

## 5. Conclusion and future perspective

RTKs such as VEGFR, EGFR and PDGFR are important therapeutic targets for human cancers, however, inhibitors of RTKs and TKs are also accompanied by a profile of adverse cardiovascular effects through unclear mechanisms. Since the cross-talk between RTKs and TRPM7 exists in multiple cell types, and TRPM7 is critically involved in both cancers and cardiovascular diseases, we suggest that TRPM7 might play a role in the cardiovascular toxicity associated with RTKI/TKIs (Figure 3). TRPM7 is ubiquitously expressed and while it is an important regulator of intracellular Mg^2+^, it also influences intarcellular Ca^2+^ homeostasis and downstream kinases and substartes that may also play a role in TRPM7-RTK/TK crosstalk. Unraveling these TRPM7-dependent processes might provide greater insights into the molecular mechanisms that underlie cardiovascular disease in patients treated with RTKI/TKIs.

## Author contributions

QL and SL wrote the manuscript. YQ and JZ provided support for the tables and figures. FR, ZZ, and RT provided guidance and corrected the manuscript. All authors contributed to the article and approved the submitted version.
